# Plasma and Visceral Organ Kynurenine Metabolites Correlate in the Multiple Sclerosis Cuprizone Animal Model

**DOI:** 10.3390/ijms26030976

**Published:** 2025-01-24

**Authors:** Helga Polyák, Zsolt Galla, Cecilia Rajda, Péter Monostori, Péter Klivényi, László Vécsei

**Affiliations:** 1 Department of Neurology, Albert Szent-Györgyi Medical School, University of Szeged, Semmelweis u. 6, H-6725 Szeged, Hungary; polyak.helga@med.u-szeged.hu (H.P.); rajda.cecilia@med.u-szeged.hu (C.R.); klivenyi.peter@med.u-szeged.hu (P.K.); 2 Preventive Health Sciences Research Group, Incubation Competence Centre of the Centre of Excellence for Interdisciplinary Research, Development and Innovation of the University of Szeged, H-6720 Szeged, Hungary; 3 Department of Pediatrics, Albert Szent-Györgyi Faculty of Medicine, University of Szeged, H-6725 Szeged, Hungary; galla.zsolt@med.u-szeged.hu (Z.G.); monostoripeter@gmail.com (P.M.); 4 HUN-REN-SZTE Neuroscience Research Group, Danube Neuroscience Research Laboratory, Hungarian Research Network, University of Szeged (HUN-REN-SZTE), Tisza Lajos krt. 113, H-6725 Szeged, Hungary

**Keywords:** cuprizone, multiple sclerosis, tryptophan metabolism, periphery, mice

## Abstract

The cuprizone (CPZ) model of multiple sclerosis (MS) is excellent for studying the molecular differences behind the damage caused by poisoning. Metabolic differences in the kynurenine pathway (KP) of tryptophan (TRP) degradation are observed in both MS and a CPZ mouse model. Our goal was to analyze the kynurenine, serotonin, and indole pathways of TRP degradation on the periphery, in the neurodegenerative processes of inflammation. In our study, mice were fed with 0.2% CPZ toxin for 5 weeks. We examined the metabolites in the three pathways of TRP breakdown in urine, plasma, and relevant visceral organs with bioanalytical measurements. In our analyses, we found a significant increase in plasma TRP, 5-hydroxytryptophan (5-HTP), and indole-3-acetic acid (IAA) levels, while a decrease in the concentrations of 3-hydroxy-L-kynurenine (3-HK), xanthurenic acid (XA), kynurenic acid (KYNA), and quinaldic acid in the plasma of toxin-treated group was found. A marked decrease in the levels of 3-HK, XA, KYNA, quinaldic acid, and indole-3-lactic acid was also observed in the visceral organs by the end of the poisoning. Furthermore, we noticed a decrease in the urinary levels of the TRP, KYNA, and XA metabolites, while an increase in serotonin and 5-hydroxyindoleacetic acid in the CPZ group was noticed. The toxin treatment resulted in elevated tryptamine and indoxyl sulfate levels and reduced IAA concentration. Moreover, the urinary para-cresyl sulfate concentration also increased in the treated group. In the present study, we showed the differences in the three main metabolic pathways of TRP degradation in the CPZ model. We confirmed the relationship and correlation between the content of the kynurenine metabolites in the plasma and the tissues of the visceral organs. We emphasized the suppression of the KP and the activity of the serotonin and indole pathways with a particular regard to the involvement of the microbiome by the indole pathway. Consequently, this is the first study to analyze in detail the distribution of the kynurenine, serotonin, and indole pathways of TRP degradation in the periphery.

## 1. Introduction

Multiple sclerosis (MS) is a neurodegenerative and chronic inflammatory disease of the central nervous system (CNS), which can be characterized by the loss of neurons and demyelination lesions in the brain and spinal cord [1,2]. Approximately 2.8 million people suffer from the disease worldwide and MS usually appears in young adulthood [3,4]. In most cases, the disease begins in a relapsing–remitting form (RRMS) and only in a small proportion of patients occurs in a progressive disease form (PMS). However, the RRMS turns into a progressive form over time [5]. Although currently available therapies are capable of modulating the relapse rate and peripheral immunity, their effectiveness is low in view of the progression of the disease and thus the worsening of the disability [5]. At the beginning of the disease, inflammatory demyelination lesions predominate with T- and B-lymphocyte predominance, while in PMS, active lesions of radial extent are present with the appearance of microglia and macrophages at their edges. In addition to these active lesions, an increasing number of inactive lesions are also present in patients with progressive MS in the CNS. Nevertheless, the changes characteristic of PMS, albeit to a lesser extent, are already present in the relapsing form of the disease. Thus, it is likely that RRMS and PMS are governed by similar pathomechanisms (for further data, see review [5]).

Several animal models are available for the study of MS, such as the experimental autoimmune encephalomyelitis (EAE) model, Theiler‘s murine encephalomyelitis virus (TMEV) infection model, the lipopolysaccharide (LPS)-induced experimental model, or the toxin-induced demyelination models, such as the lysolecithin and cuprizone (CPZ) models [6,7]. The cuprizone (CPZ) toxin-induced demyelination rodent model is often and widely used to study molecular factors and it can contribute to understanding the process of demyelination and remyelination [8]. Because the main histopathological changes in CPZ-induced lesions are very similar to the changes in the lesion pattern of MS types III and IV, which are characterized by de- and remyelination, oligodendrocyte apoptosis [9,10,11], as well as microglia and macrophage activation [12], they are also accompanied by an increased accumulation of reactive oxygen and nitrogen free radicals [13]. Thus, the CPZ model enables us to investigate the processes behind the progression of chronic white matter disease as well as demyelination and remyelination mediated by non-autoimmune processes [14], in contrast to the EAE, TMEV, and LPS models, which induce inflammatory processes [6]. CPZ is a copper chelator. It is believed to inhibit enzymes of the mitochondrial respiratory chain, thereby leading to oxidative stress, which ultimately results in apoptosis of mature oligodendrocytes, astrocyte reactivity, as well as microglia and macrophage activation [8,15]. Acute demyelination develops after 5–6 weeks of CPZ administration, which is usually used to investigate the RRMS, as rapid remyelination occurs after toxin withdrawal within a few days. However, 12 weeks of CPZ treatment results in chronic myelin damage, after which the effectiveness of remyelination is quite limited [6]. Therefore, the CPZ model is easy to apply and control and anatomically reproducible, which allows the study of de- and remyelination processes in the absence of a peripheral immune response [16].

**Figure 1:**
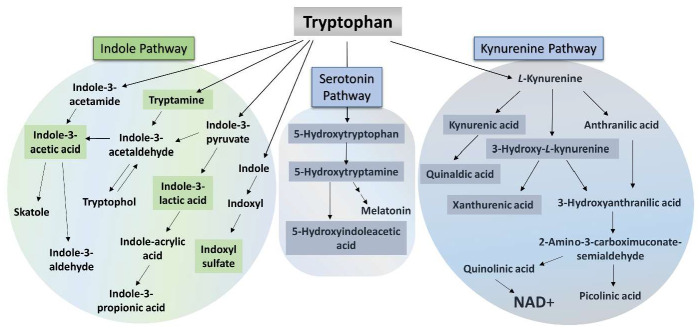
Three main pathways of tryptophan degradation. NAD+: nicotinamide adenine dinucleotide. The highlighted metabolites, in the case of which we observed discrepancies as a result of CPZ toxin treatment, in the three main pathways of TRP degradation that we investigated.

The essential amino acid tryptophan (TRP) is metabolized through the kynurenine, serotonin, and indole pathways (Figure 1). In addition to protein synthesis, TRP metabolism is related to many processes, such as nervous system function, immune response, and inflammatory processes [17]. Approximately 4–6% of TRP is catabolized to indole derivatives by the intestinal microbiome in the colon [18]. During the microbial degradation of TRP, a number of active indole derivatives are produced in the indole pathway, including indole, tryptamine, indoxyl sulfate (IS), skatole, indole-3-lactic acid (ILA), indole-3-pyruvate, indole-3-aldehyde, indole-3-acetaldehyde, indole-3-acetate, and indole-3-acetic acid (IAA) [19]. Therefore, among other compounds, TRP metabolites also play a significant role in the intestinal physiology [17]. The aryl hydrocarbon receptor (AhR) is the main molecular target of the most indole-based compounds [19]. AhR is involved in many different processes, such as metabolic diseases and neurophysiology, cell regeneration, immune responses’ regulation, carcinogenesis, or organ development [19,20,21,22,23]. The intestinal microbiome combination also contributes to the pathogenesis of several autoimmune and inflammatory diseases, including MS [24,25].

TRP can also be degraded through the serotonin pathway when 5-hydroxytryptophan (5-HTP), serotonin (5-hydroxytryptamine, 5-HT), melatonin, and 5-hydroxyindoleacetic acid (5-HIAA) are produced. Serotonin is a well-known neurotransmitter in many neuropsychiatric and neurodegenerative diseases that are involved in the regulation of various neuroendocrine functions [26]. The serotonin pathway is mediated by the gut microbiome and it is an important signaling pathway [27]. The difficulty of serotonin determination is due to its rapid metabolism by monoamine oxidase into 5-HIAA [17]. The anti-inflammatory effect of serotonin was described in EAE and MS. It also modulates gut microbiota functions, thereby indirectly playing a role in the development of neuroinflammation or autoimmunity, and plays a significant role in inflammatory diseases of the CNS, including MS [28]. A reduced concentration of 5-HIAA was described in several studies in the CSF of MS patients compared to the control group [29,30,31].

However, a significant part of dietary TRP is degraded via the kynurenine pathway (KP). The KP mostly occurs to the greatest extent in the liver where all the enzymes required for the transformation are provided, but in approximately 5–10% it can also happen extrahepatically when only certain enzymes of the pathway are available [32]. The first rate-determining step is the transformation of TRP to L-kynurenine (KYN), catalyzed by TRP-2,3-dioxygenase (TDO) and indoleamine-2,3-dioxygenases (IDOs) enzymes. Many different neuroactive metabolites can be formed from KYN, including kynurenic acid (KYNA), which may be produced by kynurenine aminotransferases (KATs), and anthranilic acid (ANA) by the kynureninase (KYNU) enzyme, while the kynurenine 3-monooxygenase (KMO) catalyzes the conversion of KYN to 3-hydroxy-L-kynurenine (3-HK). From one part, xanthurenic acid (XA) can be formed from 3-HK. On the other hand, 3-hydroxyanthranilic acid (3-HANA) can be converted to picolinic acid, quinolinic acid (QUIN), and finally, nicotinamide adenine dinucleotide (NAD+) [17]. Abnormalities of KP have been observed in many neurodegenerative and neuropsychiatric diseases, immunological illnesses, and other different diseases, including MS [17,33,34,35,36].

The two-way connection between the CNS and enteric nervous system is realized in the gut–brain axis (GBA) [37]. Studies showed that the intestinal microbiota plays a crucial role in the modulation of the GBA by influencing the production of various metabolites [37,38,39,40]. Moreover, alterations and disturbances of the gut microbiome play a significant role in GBA disorders [37,41,42,43,44]. Furthermore, by modulating the metabolism of TRP, changes in the composition of the gut microbiota influence the GBA [37,45,46]. In addition, metabolites involved in TRP breakdown, including indole derivatives, tryptamine, serotonin, or kynurenines, have a significant effect on the interaction between the GBA and the intestinal microbiota [45,47,48,49]. In recent years, the gut microbiota has appeared as a new risk factor for autoimmune diseases, including MS [24]. Additionally, in rodent models of MS, during the investigation on the EAE and CPZ models, significant microbial changes were detected as a result of the treatments, according to the progression of the disease [1].

In our previous studies, we reported decreased KYNA, XA, and 3-HK and increased TRP concentration in plasma. In addition, we noticed decreased 3-HK and ANA as well as an increased TRP level in certain brain regions at the end of the fifth week of the CPZ treatment [50,51]. Therefore, in this study, we extended our investigation to the indole pathway of TRP breakdown by the gut microbiome in order to map the relationships and differences among the kynurenine, serotonin, and indole pathways of TRP metabolism in the periphery. On the one hand, consider that alterations in the homeostasis of the gut microbiome can be found in several diseases [52]. Additionally, many lines of evidence point to the significant role of the gut–brain bidirectional communication, which may contribute to the pathogenesis and progression of neurodegenerative and neuropsychiatric disorders [53]. On the other hand, we performed a broader analysis in the periphery, since several pathomechanisms affecting visceral organs may be associated with MS, too, including cardiovascular dysfunction, renal and respiratory diseases, or abnormalities of liver function [54,55,56,57]. The pathological deviations of these visceral organs can also be linked with alterations in the kynurenine pathway of TRP breakdown [58,59,60,61]. Based on these results, due to the systemic toxic effect of CPZ, it is possible that poisoning may affect the degradation of TRP in peripheral tissues as well.

Therefore, in this study, we examined the distribution of the amount of metabolites in the three pathways of TRP breakdown in the periphery, including in the urine, plasma, and tissues from visceral organs at different times of CPZ treatment. From the consideration, that we find the correlations of the obtained alterations, and how they are related to neurodegenerative and other metabolic processes.

## 2. Results

### 2.1. Examination of Body Weight

**Figure 2:**
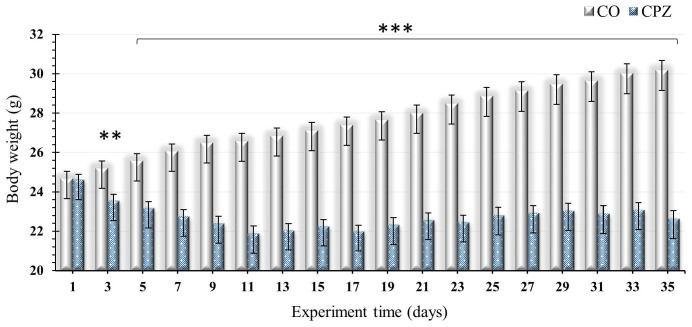
Differences in the body weight of the animals during investigation. The control group is represented by gray bars, while CPZ-treated group is represented by blue bars in the demyelination period. CO: control group; CPZ: cuprizone-treated group, **: *p* < 0.01 vs. CO, ***: *p* < 0.001 vs. CO. The data are presented as mean ± SEM.

At the beginning of the intoxication, we noticed a significant decrease in the body weight of the CPZ-treated group compared to the CO group; these differences between the groups remained unchanged and became even more marked at the end of the CPZ treatment (Figure 2).

### 2.2. Bioanalytical Measurement of Tryptophan Metabolites

**Figure 3:**
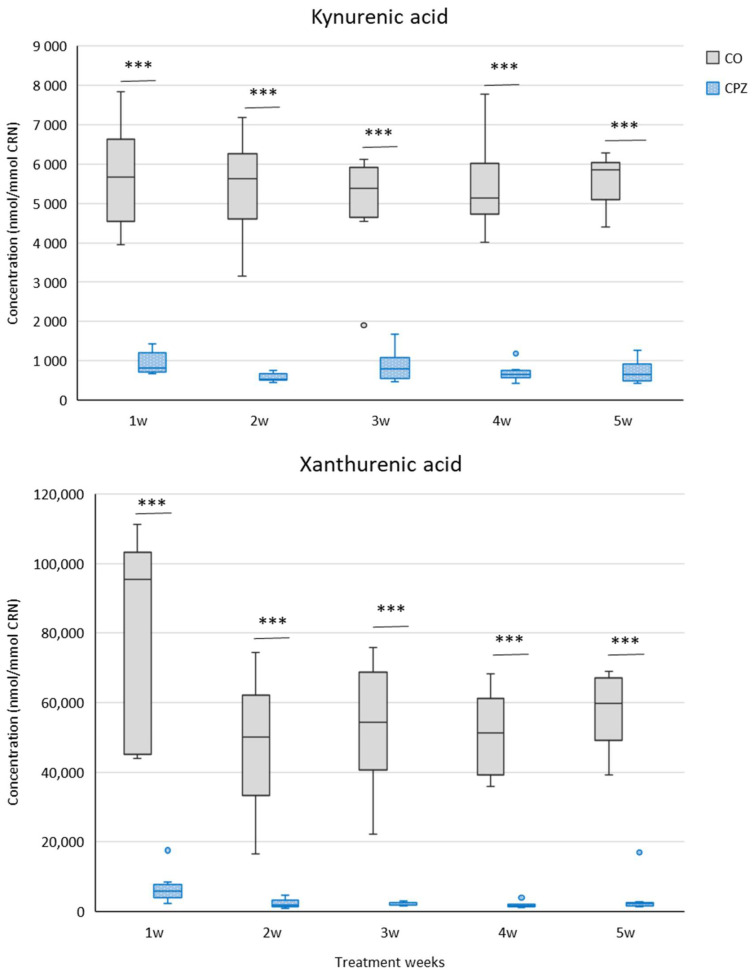
Alterations of kynurenic acid (KYNA) and xanthurenic acid (XA) concentrations during intoxication. The KYNA and XA levels were significantly reduced in urine of the CPZ-treated group during the entire period of poisoning. CO: control group, CPZ: cuprizone-treated group, w: week, ***: *p* < 0.001 vs. CO.

**Figure 4:**
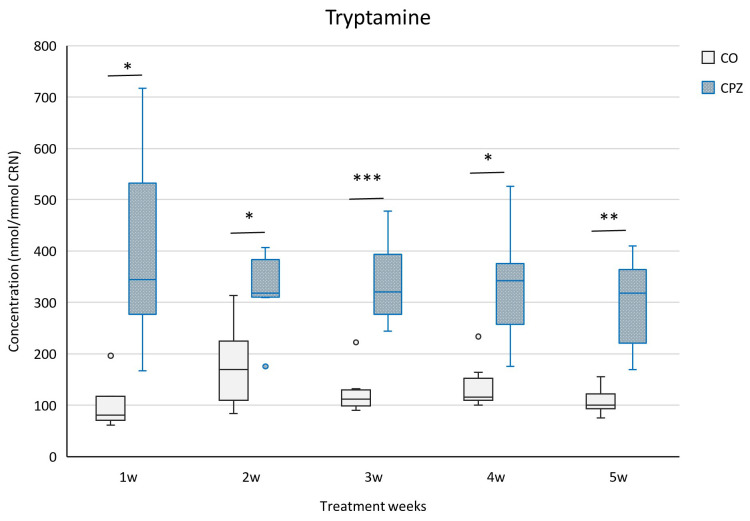
Changes in urinary tryptamine levels during treatment. The concentration of tryptamine in the urine of the toxin-treated group increased, indicating poisoning. CO: control group, CPZ: cuprizone-treated group, w: week, *: *p* < 0.05 vs. CO, **: *p* < 0.01 vs. CO, ***: *p* < 0.001 vs. CO.

During our investigation, we applied UHPLC-MS/MS bioanalytical measurements to examine the concentration changes in various metabolites produced in the indole, serotonin, and kynurenine pathways of TRP breakdown in urine samples at the end of each treatment week of CPZ poisoning, as well as in plasma at the end of the experiment. During the examination of the urine samples, we observed a significant decrease in KYNA and XA (Figure 3) concentrations as well as an increased tryptamine (Figure 4) level in the toxin-treated group during the first week of CPZ poisoning; these differences remained until the end of the treatment.

**Figure 5:**
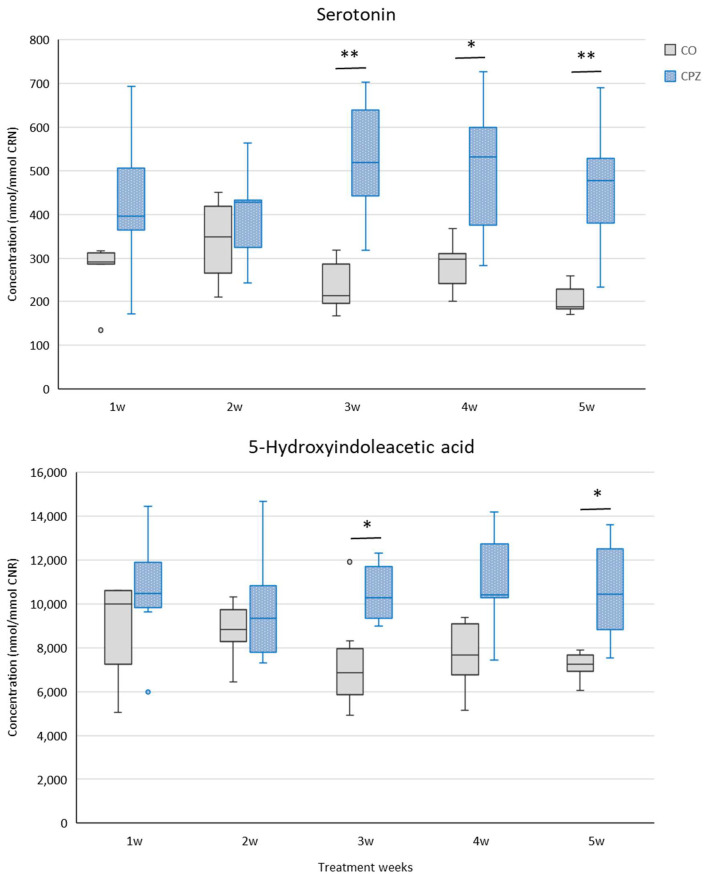
Increased serotonin and 5-hydroxyindoleacetic acid (5-HIAA) levels in the CPZ-treated group. By the third week of the intoxication, the concentrations of serotonin and 5-HIAA in the urine of the CPZ group were significantly higher compared to the CO group; these differences were also observed in the fifth week of treatment. CO: control group, CPZ: cuprizone-treated group, w: week, *: *p* < 0.05 vs. CO, **: *p* < 0.01 vs. CO.

From the third week, the levels of serotonin and 5-HIAA showed differences between the CPZ and CO groups (Figure 5).

**Figure 6:**
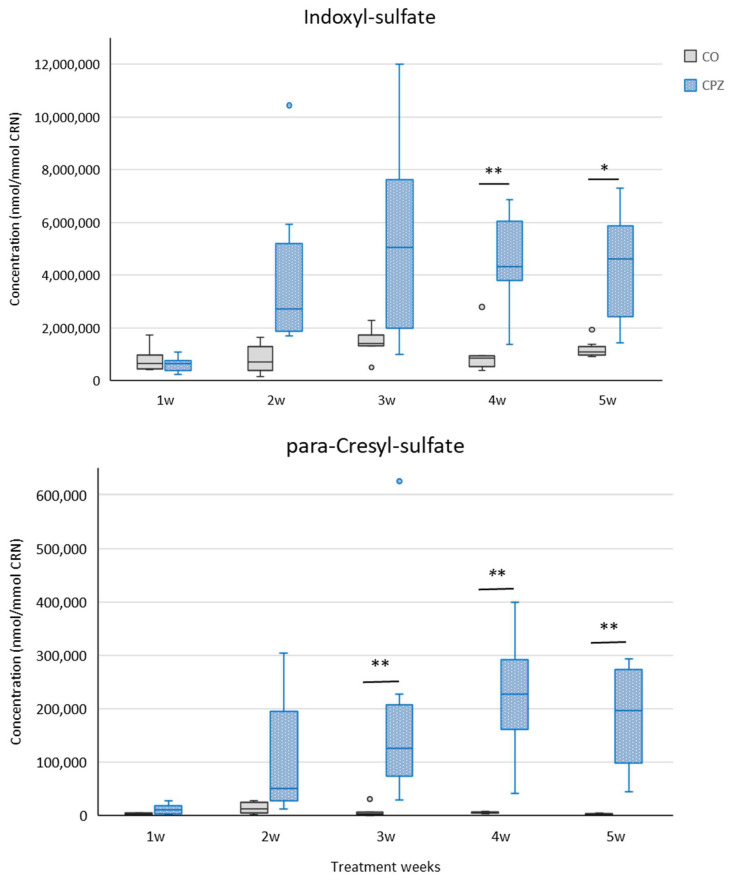
Alterations of indoxyl sulfate (IS) and para-cresyl sulfate (pCS) concentrations during CPZ poisoning. In the fourth week of treatment, the urinary IS level of the CPZ group was significantly increased compared to the CO group, while the level of pCS already differed markedly between the groups in the third week of intoxication. CO: control group, CPZ: cuprizone-treated group, w: week, *: *p* < 0.05 vs. CO, **: *p* < 0.01 vs. CO.

In the third week of treatment, in addition to the mentioned metabolites, an increase in the level of urinary para-cresyl sulfate (pCS) was noticed, while from the fourth week of intoxication, an elevated concentration of IS was observed in the CPZ-intoxicated group compared to the CO group (Figure 6). At the end of intoxication, the plasma IS and pCS levels also increased in the CPZ-treated group; however, these differences were not significant.

**Figure 7:**
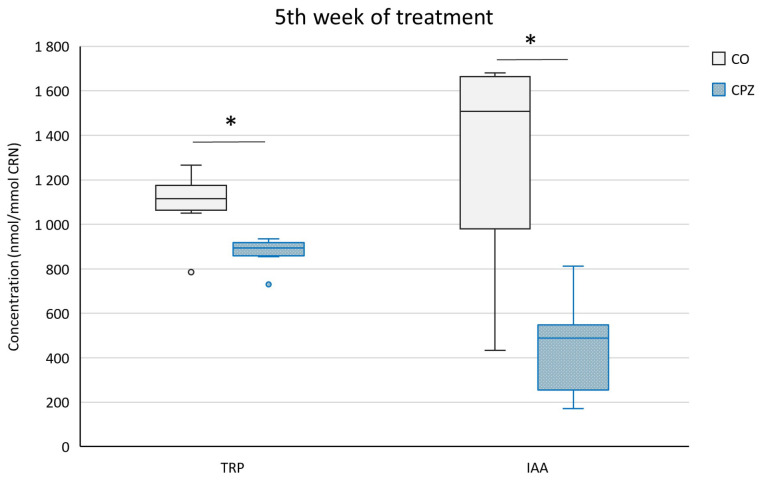
Decreased tryptophan (TRP) and indole-3-acetic acid (IAA) levels at the end of treatment. The concentrations of TRP and IAA were markedly reduced in the urine of the toxin-treated group in the fifth week of poisoning. CO: control group, CPZ: cuprizone-treated group, w: week, *: *p* < 0.05 vs. CO.

At the end of the fifth week of treatment, in addition to the aforementioned metabolite differences, even the TRP and IAA concentrations showed significant distinctions in the urine between the CPZ and CO groups (Figure 7).

**Figure 8:**
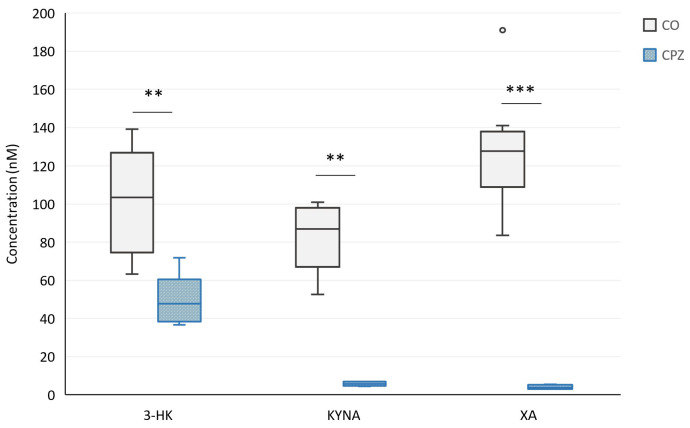
Alterations of kynurenine metabolites in plasma during intoxication. In the fifth week of poisoning, the concentrations of 3-hydroxy-L-kynurenine (3-HK), kynurenic acid (KYNA), and xanthurenic acid (XA) were significantly decreased in the CPZ-treated group. CO: control group, CPZ: cuprizone-treated group, w: week, **: *p* < 0.01 vs. CO, ***: *p* < 0.001 vs. CO.

**Figure 9:**
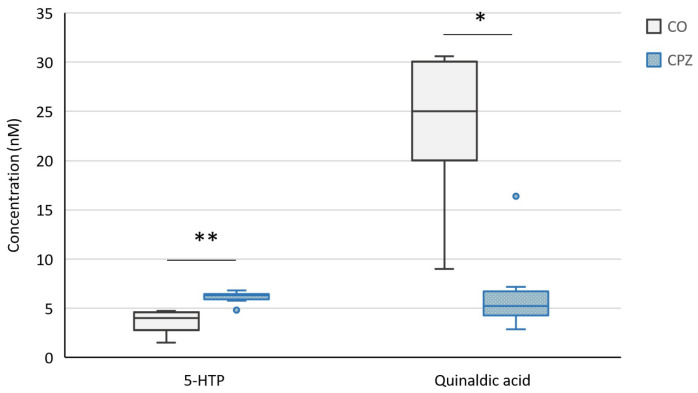
Changes in plasma 5-hydroxytryptophan (5-HTP) and quinaldic acid levels at the end of treatment. After 5 weeks of intoxication, the concentration of 5-HTP increased in the CPZ-treated group while the level of quinaldic acid decreased in plasma. CO: control group, CPZ: cuprizone-treated group, w: week, *: *p* < 0.05 vs. CO, **: *p* < 0.01 vs. CO.

**Figure 10:**
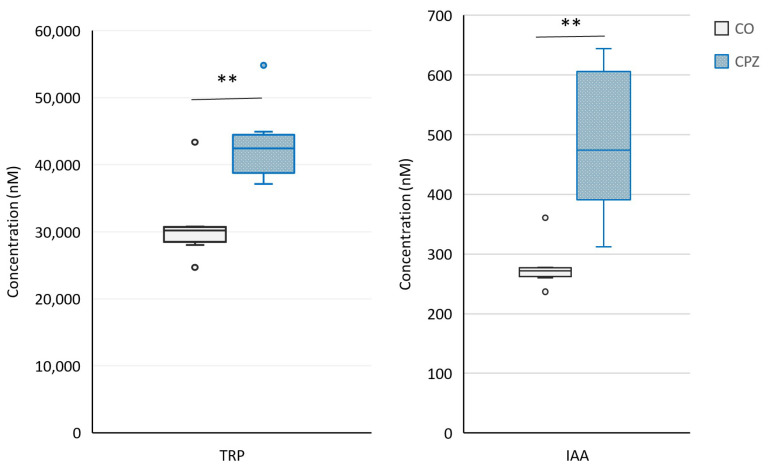
Increased tryptophan (TRP) and indole-3-acetic acid (IAA) concentrations in the fifth week of treatment. At the end of the poisoning, the plasma levels of TRP and IAA were remarkably elevated in the CPZ group compared to the CO group. CO: control group, CPZ: cuprizone-treated group, w: week, **: *p* < 0.01 vs. CO.

Moreover, at the end of the 5-week poisoning, in the case of plasma samples, we observed a significant decrease in the concentrations of 3-HK, KYNA, XA (Figure 8), and quinaldic acid (Figure 9) as well as an increase in the levels of 5-HTP (Figure 9), TRP, and IAA in the CPZ-treated group compared to the CO group (Figure 10).

**Figure 11:**
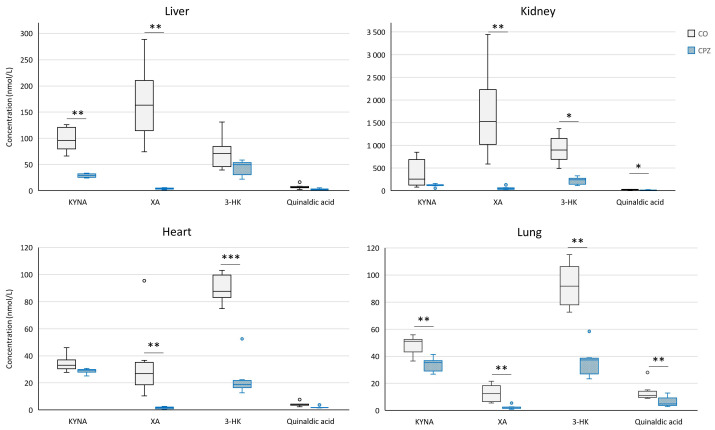
Decreased kynurenine metabolites in the visceral organs at the end of the poisoning. In the fifth week of treatment, the kynurenic acid (KYNA), xanthurenic acid (XA), 3-hydroxy-L-kynurenine (3-HK), and quinaldic acid concentrations were markedly reduced in the liver, kidney, heart, and lungs. CO: control group, CPZ: cuprizone-treated group, w: week, *: *p* < 0.05 vs. CO, **: *p* < 0.01 vs. CO, ***: *p* < 0.001 vs. CO.

**Figure 12:**
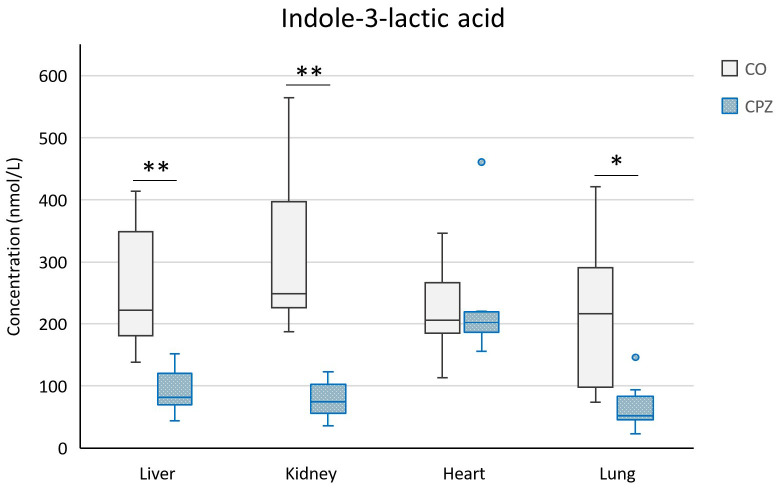
Reduced indole-3-lactic acid (ILA) level in the visceral organs of the CPZ group. As a result of poisoning, the ILA concentration was significantly decreased in the liver, kidney, and lungs as well as in the heart, where the difference was not significant. CO: control group, CPZ: cuprizone-treated group, w: week, *: *p* < 0.05 vs. CO, **: *p* < 0.01 vs. CO.

In addition, during the bioanalytical analysis of the visceral organs, we observed a marked decrease in the levels of KYNA, XA, 3-HK, and quinaldic acid (Figure 11) as well as ILA (Figure 12) in the liver, kidney, heart, and lungs of the animals treated with the CPZ toxin.

## 3. Discussion

In the present study, we examined in detail the differences in the metabolic pathways of TRP breakdown in the periphery at different times of intoxication and at the end of it. Given that, in our previous studies, we confirmed the KP role of TRP metabolism in the CPZ rodent model of MS, in accordance with the progress of the toxin treatment and the severity of the damage [50,51]. As a continuation of this investigation, we further analyzed the distribution of the kynurenine, serotonin, and indole metabolic pathways of TRP degradation in the urine, plasma, and tissues from visceral organs with bioanalytical measurements, thus further expanding our knowledge in the CPZ mouse model. We were curious to see what alterations TRP metabolism showed in the periphery, following the differences observed in the CNS [51].

In our study, as the toxicity progressed and worsened, the body weight of the CPZ-treated mice gradually decreased; these deviations are in line with our previous results [51]. In our investigation, because of the treatment, we found decreased concentrations of KYNA, XA, TRP, and IAA in the urine as well as increased levels of tryptamine, serotonin, 5-HIAA, IS, and pCS in the CPZ-treated group compared to the CO. In the case of plasma, at the end of the 5-week intoxication, the KYNA, XA, 3-HK, and quinaldic acid concentrations of the CPZ group decreased markedly, while the levels of 5-HTP, TRP, and IAA elevated during poisoning. Regarding the visceral organs, a significant decrease in the concentrations of KYNA, XA, 3-HK, and quinaldic acid was experienced in the CPZ toxin-treated group compared to the CO group; these data confirmed the results of the plasma sample. Similarly, the level of ILA was also markedly lower in the organs as a result of the effect of the poisoning.

Based on our present research results, it seems that during CPZ intoxication, the TRP breakdown shifts towards the serotonin and indole pathways while the kynurenine pathway takes a back seat, which can be explained by the reduced levels of some kynurenine metabolites seen in the periphery. The increased TRP plasma level and its reduced concentration in urine during the fifth week of intoxication may indicate that, as the poisoning progresses, the body tries to limit the excretion of the essential amino acid, thereby maintaining the activity of the metabolic pathways associated with TRP degradation. Toxin treatment may also affect the absorption and utilization of nutrients, including TRP, and thus may impact body weight. The KYNA and XA, due to their neuroprotective properties, may be used up during poisoning, thereby reducing the extent of damage and promoting recovery. A reduced KYNA concentration was described in several neurodegenerative diseases, including Alzheimer’s disease, Parkinson’s disease, MS, and epilepsy, among others [36]. According to the literature data, KYNA levels decrease in the progressive form of MS [62]. Rejdak et al. also observed a decreased KYNA concentration in MS patients in remission [63]. Furthermore, reduced KYNA levels were reported in autism, depression, and schizophrenia [64,65,66]. The reduced level of quinaldic acid may result from a low concentration of KYNA, as it is its metabolite. The reduced concentration of the neuroactive 3-HK may also be explained by its beneficial role. Since it does not function as a toxic metabolite in all cases, 3-HK maybe contribute to the regulation and protection of the redox balance of the brain tissue and it can also act as a scavenger [51,67,68,69]. A recent study reported a decreased 3-HK level in MS patients, which was associated with microglial activity [70]. In addition, reduced plasma and serum concentrations of 3-HK were also described in patients with major depressive disorder and schizophrenia [71,72].

The data from our present study confirm the results of our previous studies, in which we noticed reduced kynurenine metabolites’ concentrations and an increased TRP level in the periphery during CPZ treatment [51]. However, we did not find any changes in the concentrations of certain metabolites, such as picolinic acid, 3-HANA, or QUIN. QUIN is a neurotoxic metabolite that shows alterations in several neurodegenerative diseases [36]. It may be assumed that changes in the levels of these metabolites may be influenced by the duration of toxin treatment and, thus, the degree of damage; so, perhaps a longer CPZ administration is needed to detect the differences.

Furthermore, studies showed that IDO enzymes play a key role in the degradation of TRP and its metabolites, including 5-HTP, serotonin, and melatonin [26]. The gut microbiome also has a significant impact on TRP metabolism via the serotonin and kynurenine pathways [27]. Moreover, serotonin can also enter the kynurenine pathway through the IDO1 enzyme [73]. Studies suggest that metabolites from the two pathways interact with each other by affecting TRP availability or the function of certain enzymes and receptors [73,74]. In addition, both serotonin and kynurenine have an important effect on the gut microbiome and there is a complex relationship between these pathways. However, there is still relatively little information available about the interaction between these two pathways of TRP metabolism. Due to these close correlations, it is very important to investigate the serotonin and kynurenine pathways together in both physiological and various pathophysiological processes [73]. Several studies also found a correlation between elevated serotonin levels and improved outcomes in patients with MS, while animal studies suggested that elevated serotonin concentration may exert immunomodulatory effects and slow progression. These findings and data seem promising for studies and therapeutic options targeting the serotonergic system [75].

The available TRP level in the body can result in significant differences in serotonin and melatonin levels and, thus, can be linked to the pathomechanisms of various neuropsychiatric disorders [26]. Studies suggest that alterations in serotoninergic neurotransmission are associated with the pathophysiology of psychiatric diseases [26]. Psychiatric comorbidities, such as depression, anxiety, personality and bipolar disorders, as well as cognitive and personality changes, are also common in patients with MS, and these manifestations significantly affect the quality of life and progression of the disease [76]. A lower serotonin concentration was reported in MS patients, which correlated with disability [77]. Furthermore, depression in MS may be linked to inflammatory mechanisms and alterations of the serotonin and KP of TRP metabolism [78]. In patients with depression, a decrease in neuroprotective and an increase in neurotoxic metabolites were observed, which, over time, were significantly associated with the development of symptoms [79,80,81]. Moreover, inflammatory markers and IDO enzyme activity were associated in patients with depression who attempt suicide [82]. In addition, similar KP deviations can be observed in suicidality and progressive MS [78,82]. In bipolar disorders, TRP catabolism is downregulated, while in schizophrenia, a reduced TRP plasma concentration was reported, which may be related to an increased conversion of the TRP to kynurenine metabolites [26]. Additionally, lower peripheral serotonin levels were described in patients with major depressive disorder, while a reduced KYNA concentration may play a role in severe cognitive symptoms in depressed patients [26].

Abnormalities in the metabolic pathways of TRP degradation were also reported in some diseases, such as irritable bowel syndrome, chronic kidney disease, obesity, and anorexia nervosa or bulimia, among others [83]. Recently, the regulation of the gut microbiome and its role in various disorders have increasingly become the focus of research. Studies highlighted the influences of the gut microbiome on host immunity in various neuroinflammatory diseases [84,85,86]. Furthermore, deviations and changes in the microbiome were also described in several neurodegenerative diseases, and these alterations are associated with disease progression in MS. In addition, some researchers drew attention to the relationship between TRP metabolism and the gut–brain axis [1,24,25,37,87]. The correlations between the gut microbiome and different diseases further emphasize the crucial role of metabolites involved in the indole pathway of TRP degradation.

Tryptamine is formed from TRP via the activation of the catabolic enzyme TRP decarboxylase by the gut microbiome [88,89]. Based on studies, tryptamine plays a beneficial role in the EAE model, relieving the clinical symptoms of EAE by limiting neuroinflammation through overactivation of the AhR [86]. Furthermore, research suggests that microbial tryptamine can influence gut flora by affecting gut motility and the enteric nervous system [27,86,89,90]. In EAE mice, Dopkins et al. described a marked increase in the level of the anti-inflammatory butyrate metabolite of bacterial origin in response to tryptamine treatment [86]. Tryptamine also has an inhibitory effect on the IDO1 enzyme, thus regulating the immune responses [27,91]. In addition to activating the AhR, it modulates the immune function of the intestinal system [92]. Also, tryptamine may promote serotonin release [93], and changes in serotonin concentration can influence bowel movements [89,94]. However, based on studies, as a result of the increased TRP concentration, the tryptamine level in the serum and gut was elevated remarkably [92,95], thereby transforming and altering the distribution of TRP metabolites by microbes, impacting the physiological functioning of the gut microbiome [27].

Several studies highlighted the relationship between the change in the composition of the gut microbiome and the pathogenesis of various neurological disorders [96,97,98]. An altered gut microbiome was described in patients with MS compared to the controls [99,100,101,102]. Furthermore, bacterial toxins reaching the CNS via the bloodstream [101,102,103,104] can even cause the cell death of oligodendrocytes [105,106,107]. The aerobic bacteria with urease activity are liable for the formation of protein-bound uremic toxins, including IS and pCS [108,109,110,111]. The amount of IS and pCS increased significantly in our investigation because of the CPZ treatment. The elevated concentration of these metabolites can cause a number of harmful effects, such as cell damage, immunosuppression, as well as chronic inflammation [110,112,113]. IS contributes to the elevated concentrations of certain anti-inflammatory cytokines [114]. In addition, it exerts a harmful effect on neurons, astrocytes, and glial cells, induces oxidative stress, and promotes the creation of a neurotoxic environment, thus playing a key role in neurodegeneration [115,116]. The pCS affects the immune system by suppressing the functions of macrophages [117]. Furthermore, these metabolites can also promote, among others, the production of reactive oxygen species (ROS) and the inhibition of the antioxidant superoxide dismutase (SOD) enzyme [110,114]. In a study among patients living with MS, bacterial IS and pCS were identified as potential neurotoxic metabolites. The amount of these metabolites decreased as result of a disease-modifying therapy, and the level of toxins correlated with the level of the neurofilament light chain [107]. In addition, bacterial neurotoxic metabolites induced axonal damage in cultured neurons [107]. The data of the mentioned studies suggest that metabolites of bacterial origin in the indole pathway of TRP breakdown have an effect on the gut–brain communication and can cause neurotoxicity in MS [107]. Moreover, in a pathological state, TRP can transform into indole derivatives [107,118,119]. IS accumulation is related to the impairment of kidney function [120]. Furthermore, alterations in the gut microbiome also affect the plasma levels of IS and pCS [120]. The elevated IS and pCS concentrations observed in our study may indicate an imbalance in the gut microbiome, during which these microbial metabolites may increase the degree of damage caused by the CPZ toxin due to their pro-oxidative properties. The increase in plasma IS and pCS concentrations, which did not result in a significant difference between the CO and CPZ groups, may confirm the suggestion that the body’s effort to remove toxins through urine proves to be effective.

Another protein-bound uremic toxin is the IAA. Studies reported that IAA promotes certain pro-inflammatory processes, as well as raising ROS production [121], thus promoting the processes of neurodegeneration [122]. IAA was related to oxidative stress, cytotoxicity, neuropathic pain, as well as cognitive impairment [111,116,121]. In addition, IAA and IS can affect the functions of and toxicity to the kidney and other organs. They activate inflammatory processes and oxidative stress, too [120]. Moreover, the reductive and oxidative imbalance of the microbial indole pathway of TRP breakdown plays a crucial role in MS [87]. A strong correlation was observed between oxidative IAA metabolite and increased MS risk and disease progression, while reduced levels of the reductive ILA metabolite can be associated with the worsening of the disorder process, as well as with a higher EDSS score in MS [87]. ILA is one of the most significantly reduced indole metabolites in MS, and a decrease in the number of microbes producing ILA was also reported among patients with MS [87,123]. Especially reduced ILA levels were announced in progressive MS [87,123]. Another study also reported a reduced serum ILA level in patients with MS; additionally, in childhood MS, the onset of the disease could be inferred from ILA levels [124]. Moreover, a lower level of ILA can result in an increased activity of inflammatory diseases in the CNS and periphery. Furthermore, IAA can induce inflammatory and oxidative stress [120,125]. In addition to that, a study reported that IAA treatment enhances the production of tumor necrosis factor-alpha [87]. Moreover, it was also described that IAA aggravated EAE [126]. IAA can cause serious cytotoxicity and hematotoxicity as well as enhancing the process of necrosis and apoptosis, among others [127,128,129]. However, based on several studies, IAA administration reduced the appearance and severity of the disease in a mouse model as well as improving the gut microbiome balance and inflammatory processes [25]. Furthermore, other protective effects were also reported in various studies [120,130,131]. In our present investigation, we noticed an increased IAA plasma concentration and decreased IAA urine level, as well as a decreased ILA level in the visceral organs in the CPZ group at the end of intoxication; the results in this case may be related to the damage caused by poisoning and changes in the intestinal microbiome.

Serotonin participates in immunomodulation, mediates bowel movements, as well as influences the processes of autoimmunity and neuroinflammation [28,132]. In addition, studies suggest that serotonin may have both pro- and anti-inflammatory functions, too [28]. A recent study reported different serotonin and 5-HIAA serum concentrations between the control and CPZ-treated groups [133]. Moreover, several studies described the beneficial effect of selective serotonin reuptake inhibitors in MS and EAE as well as in other autoimmune diseases [28]. Alterations of the gut microbiome can cause a disturbance in the balance of serotonin [120]. Furthermore, the microbiome determines the serotonin concentration of the entire body, hindering the TRP kynurenine conversion as a competing process [28,134]. Hence, we assume that the poisoning of CPZ by feeding understandably has a significant effect on the gut microbiome, because of which the kynurenine pathway of TRP degradation is downregulated while the serotonin pathway is activated; these may lead to an increased level of metabolites in the pathway.

The intestinal system has a direct connection with the brain via the nervus vagus, and the gut microbiome has a close relationship with the nervous and immune systems [135,136]. As a result of the strong connection, the interactions of the gut–brain axis play an important role not only in the physiological functions of the body but also in the pathogenesis of diseases, including MS [28]. A large number of microbial strains participate in the complex metabolism of TRP and its transformation into indole derivatives [18,137]. Alterations in the gut microbiome were described not only in MS but in its EAE- and CPZ- induced animal models, too [1,138]. Consequently, it is not surprising that the microbial differences caused by CPZ poisoning also affect the degradation of TRP by the gut microbiome. Metabolites from the degradation participate in the regulation of microglia by astrocytes in the CNS [48,120]. Certain indole compounds can regulate the AhR activity, therefore regulating several AhR-linked functions [120]. Indole metabolites may be considered as so-called biomarkers in many disorders, since their changes in concentration can be associated with the pathophysiology of various diseases [120]. Based on the aforementioned research results, an analysis and exploration of the role of TRP breakdown pathways in physiological and pathological processes are crucial in order to gain an even wider understanding of the pathomechanisms of diseases and the effectiveness of therapeutic approaches.

The authors acknowledge the limitations of the study and the use of animal models. MS is a highly heterogeneous disease with a complex pathomechanism and diverse symptoms. The CPZ mouse model can only partially imitate the demyelination and remyelination processes occurring in MS [16]. Nonetheless, the use of animal models is undoubtedly essential to map in detail different aspects of the molecular mechanisms underlying the pathomechanism of the disease [6].

Furthermore, our data shed light on the involvement of indole metabolites of microbial origin and, consequently, the gut–brain axis, too. Additionally, these results may con-tribute to a broader analysis of other metabolic pathways that may be directly or indirectly linked to TRP metabolism, as there is still little data available in this field.

Nevertheless, this is a pioneering study that comprehensively investigated the distribution of the kynurenine, serotonin, and indole pathways of TRP degradation in the periphery in a CPZ-toxin induced demyelination animal model of MS. Moreover, our analyses so far confirmed each other with a robust pattern of metabolic abnormalities. Additionally, our experimental data are remarkably consistent, which proved to us that it is important and useful to examine the periphery, and it is not necessary to analyze different samples simultaneously; in case of obstruction, even examining one sample type may be sufficient. In the long term, transferring these examination options to clinical practice may present a much more practical solution in terms of follow-up as well as disease monitoring and progression.

## 4. Materials and Methods

### 4.1. Animal Experiments and Sample Collection

Eight-week-old C57BL/6J male mice were used (n = 24) in our investigation. The animals were bred and maintained under standard laboratory conditions with 12 h‒12 h light/dark cycle at 24 ± 1 °C and 45‒55% relative humidity in the Animal House of the Department of Neurology, University of Szeged. The investigations were in accordance with the Ethical Codex of Animal Experiments and were approved by the Ethics Committee of the Faculty of Medicine, University of Szeged, and the National Food Chain Safety Office with a permission number of XI/1101/2018. The experiment was performed as previously described in our previous studies [50,51]. Briefly, the animals were housed in groups of 5 in polycarbonate cages (530 cm^3^ floor space). Before starting the experiment, animals were acclimated to ground, standard rodent chow for 2 weeks, and the weight of the animals was measured every-other day.

**Figure 13:**
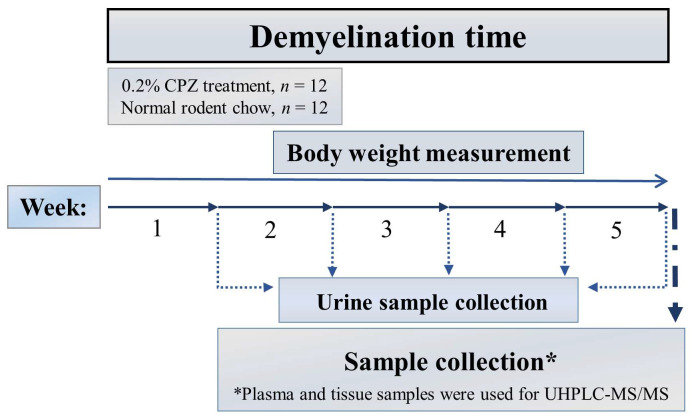
Timeline of the experimental procedure used. CPZ: cuprizone group; n: represents the number of animals used in one group; UHPLC-MS/MS: ultra-high-performance liquid chromatography-tandem mass spectrometry; numbers (1–5) in the figure: experimental weeks (week 1 to week 5); * Plasma, liver, kidney, heart, and lung samples were used for bioanalytical measurements both from the CO and the CPZ-treated groups.

Half of the experimental animals (n = 12) were treated for 5 weeks with 0.2% CPZ (bis-cyclohexanone–oxaldihydrazone; Sigma-Aldrich, St. Louis, MO, USA) mixed into a ground, standard rodent chow. As control (CO) group, age- and weight-matched animals were used (n = 12); they also had free access to water and rodent chow. During the treatment period (demyelination phase), urine samples were collected from the animals (n = 12, 6 control and 6 CPZ-treated animals) at the end of each CPZ treatment week. At the end of the fifth week of treatment period, all the animals were terminated (n = 24, 12 control and 12 CPZ-treated animals), as follows (Figure 13).

The animals were terminated based on our previous studies [50,51]. In summary, the mice were anesthetized with intraperitoneal 4% chloral hydrate (10 mL/kg body weight). For further studies, mice (CO: n = 12, CPZ: n = 12) were perfused transcardially with artificial cerebrospinal fluid (aCSF). Blood samples were taken from the left heart ventricle into Eppendorf tubes with and without disodium ethylenediaminetetraacetate dihydrate, and the plasma samples were separated by centrifugation (3500 rpm for 10 min at 4 °C). The visceral organs, namely, the liver, kidney, heart, and lungs, were removed from the animals. All samples were removed on ice and stored at −80 °C until further use.

### 4.2. Ultra-High-Performance Liquid Chromatography with Tandem Mass Spectrometry (UHPLC-MS/MS) Measurement

Urine and plasma samples were prepared and measured according to previously published methodologies [139,140] using ultra-high-performance liquid chromatography-tandem mass spectrometry (UHPLC-MS/MS). Tissue samples were prepared [51] and measured [139,140] according to previously published methodologies using UHPLC-MS/MS. The extraction solvent of the tissues was ultrapure water + 0.2% formic acid. The ratio of extraction solvent and tissue was 3:1. The precipitating solvent was 100% acetonitrile, and the ratio of tissue samples and the precipitating solvent was 1:19.2. MRM transition of Indole-3-lactate was 206.1/188.1 using 50 V as declustering potential and 13 V as collision energy, retention time: 12.0 min.

MRM transition of indoxyl sulphate was 211.9/131.9 using −50 V as declustering potential and −25 V as collision energy, retention time: 11.8 min. MRM transition of para-cresyl sulfate was 186.9/107.0 using −50 V as declustering potential and −26 V as collision energy, retention time: 12.7 min.

### 4.3. Statistical Analysis

The statistical analyses were carried out with the IBM SPSS Statistics 28.0 software (SPSS Inc., Chicago, IL, USA). For the statistical analysis of body weight, two-way repeated-measures analysis of variance (ANOVA) was used. Pairwise comparisons of group means were based on the estimated marginal means with Sidak or Tamhane’s T2 post hoc test with adjustment for multiple comparisons. Regarding the UHPLC-MS/MS measurements, after checking for its assumptions (checking for outliers, Shapiro and Levene tests), in the case of the urine samples, we applied two-way repeated-measures ANOVA, while for the tissue and plasma samples, we performed independent samples t-test, with estimated marginal means post hoc tests to determine significance between the groups. In the case of the assumptions not being met, we applied non-parametric statistics (Mann–Whitney U test). We rejected null hypotheses when the corrected *p* level was <0.05, and, in such cases, the differences were considered significant.

## Data Availability

The data presented in this study are available on request from the corresponding author.

## Abbreviations

ANA	Anthranilic acid
CO	Control
CNS	Central nervous system
CPZ	Cuprizone
DEM	Demyelination
5-HIAA	5-hydroxyindoleacetic acid
5-HTP	5-hydroxytryptophan
3-HK	3-hydroxy-L-kynurenine
HPLC	High-performance liquid chromatography
IAA	Indole-3-acetic acid
IDO	Indoleamin-2,3-dioxygenase
ILA	Indole-3-lactic acid
IS	Indoxyl sulfate
KAT	Kynurenine aminotransferase
KMO	Kynurenine 3-monooxygenase
KP	Kynurenine pathway
KYN	Kynurenine
KYNA	Kynurenic acid
UHPLC-MS/MS	Ultra-high-performance liquid chromatography-tandem mass spectrometry
MS	Multiple sclerosis
NAD+	Nicotinamide adenine dinucleotide
pCS	para-cresyl sulfate
PICA	Picolinic acid
PMS	Progressive multiple sclerosis
ROS	Reactive oxygen species
RRMS	Relapsing–remitting multiple sclerosis
TDO	Tryptophan 2,3-dioxygenase
TRP	Tryptophan
XA	Xanthurenic acid
QUIN	Quinolinic acid
